# Supplementary material for: Semantic Variant Primary Progressive Aphasia: Practical Recommendations for Treatment from 20 Years of Behavioural Research
Source: Brain Sci. 2021 Nov 23;11(12):1552. doi: 10.3390/brainsci11121552 (PMC8699306; doi:10.3390/brainsci11121552)
Supplement: Supplementary file 1 [file brainsci-11-01552-s001.zip › brainsci-1408287-supplementary26Nov.pdf]

brain sciences

Review

Semantic Variant Primary Progressive Aphasia: Practical Recommendations for Treatment from 20 Years of Behavioural Research

Aida Suárez-González [1,*,†], Sharon A Savage [2], Nathalie Bier [3,4], Maya L. Henry [5,6], Regina Jokel [7,8], Lyndsey Nickels [9] and Cathleen Taylor-Rubin [9,10]

1 Dementia Research Centre, UCL Queen Square Institute of Neurology, University College London, London postcode, UK
2 School of Psychological Sciences, College of Engineering, Science and Environment, The University of Newcastle, Callaghan postcode, NSW, Australia; sharon.savage@newcastle.edu.au
3 CIUSSS-CSMTL site Centre de Recherche de l'Institut Universitaire de Gériatrie de Montréal, Quebec postcode, QB; Canada; nathalie.bier@umontreal.ca
4 Université de Montréal, Quebec postcode, QB, Canada
5 Department of Speech, Language, and Hearing Sciences, The University of Texas at Austin, zip code. Austin, TX, USA; maya.henry@austin.utexas.edu
6 Dell Medical School, The University of Texas at Austin, Austin, TX, USA
7 Rotman Research Institute, Baycrest Health Sciences, Toronto postcode, ON, Canada; rjokel@research.baycrest.org
8 Department of Speech-Language Pathology, University of Toronto, Toronto postcode, ON, Canada
9 School of Psychological Sciences, Macquarie University, Sydney postcode, NSW, Australia; lyndsey.nickels@mq.edu.au (L.N.); Cathleen.Taylor@health.nsw.gov.au (C.T.-R.)
10 Department of Speech Pathology, Uniting War Memorial Hospital, South Eastern Sydney Local Health District War Memorial Hospital, Waverley postcode, NSW, Australia
* Correspondence: aida.gonzalez@ucl.ac.uk
† Current Address: Aida Suárez-González, UCL Queen Square Institute of Neurology, Dementia Research Centre, box 16, 8-11 Queen Square, London WC1N 3BG, UK.

Citation: Suárez-González, A.; Savage, S.A.; Bier, N.; Henry, M.L.; Jokel, R.; Nickels, L.; Taylor-Rubin, C. Semantic Variant Primary Progressive Aphasia: Practical Recommendations for Treatment from 20 Years of Behavioural Research. *Brain Sci.* **2021**, *11*, 1552. https://doi.org/10.3390/brainsci11121552

Academic Editor(s): Jordi A. Matias-Guiu

Received: 20 September 2021
Accepted: 16 November 2021
Published: date

Publisher's Note: MDPI stays neutral with regard to jurisdictional claims in published maps and institutional affiliations.

Copyright: © 2021 by the authors. Submitted for possible open access publication under the terms and conditions of the Creative Commons Attribution (CC BY) license (http://creativecommons.org/licenses/by/4.0/).

Supplementary 1 (English). Semantic Variant Primary Progressive Aphasia (svPPA): evidence-based recommendations for therapy and management

The following clinical recommendations have been produced according to the best available evidence in July 2021 as part of the paper: Aida Suárez-González, Sharon A. Savage, Nathalie Bier, Maya Henry, Regina Jokel, Lyndsey Nickels and Cathleen Taylor-Rubin. Semantic Variant Primary Progressive Aphasia: practical recommendations for treatment from 20 years of behavioural research. Brain Sciences, 2021.

1.1. Word and conceptual relearning therapies

<u>*Who?*</u> All people with svPPA able to engage in therapy (e.g., without severe behavioural disturbances) are candidates for treatment regardless of disease severity. svPPA variants with left and right hemisphere dominant atrophy show different clinical profiles and therapy should be tailored accordingly.

<u>*What?*</u> Greater therapy gains are obtained with treatment material based on words/concepts that are personally familiar (e.g., your own toaster instead of a standard toaster), belonging to daily vocabulary and retaining residual semantic knowledge (i.e., the person can still understand the concept).

<u>*How?*</u>

- 20–60 min of daily (or every other day) practice comprising 15–30 items per session should produce visible gains within the first month of practice or earlier.
- Anomia therapy can improve retrieval of words with spared semantic knowledge. However, when this approach is used with concepts where

semantic knowledge is degraded, it can lead to rote-learning (rigid and context-dependent word recall). To improve the chances of flexible learning and generalisation for these items, strategies that target the semantic nature of anomia are recommended (e.g., leveraging episodic and autobiographical memory and semantic features such as use and location).
- Tele-therapy and self-administered home-programmes have proved feasible in svPPA. Gains are comparable to face-to-face therapy.

Maintenance

Gains can be maintained for up to 6 months in mild cases (without need for revision); Low intensity revision of re-learned vocabulary is advised every 2 months for mild-moderate svPPA and every 2–3 weeks for severe svPPA.

Generalisation

Generalisation remains limited in svPPA for untrained items. Use of trained words outside of the therapy context has anecdotally been reported in some cases. The choice of therapy programme may influence generalisation.

Prophylaxis

Regular practice of words/concepts that the person can still name might help slow progression of semantic loss and anomia for those items.

1.2. Assistive technologies and ADL training

Portable technologies and training of ADL might support independence in everyday living in people with svPPA, as suggested in the few studies conducted to date.

1.3. Support groups and educational programmes

Educational and peer support groups are valuable adjuvants to therapy yielding benefits for mental health, management of disease's symptoms and quality of life.

Supplementary 2 (French). Variante sémantique de l'aphasie progressive primaire (vsAPP): indications fondées sur les données probantes pour le traitement et la prise en charge

Les indications cliniques suivantes ont été produites selon les meilleures évidences scientifiques disponibles en juillet 2021 dans le cadre du document suivant : *Aida Suárez-González, Sharon A. Savage, Nathalie Bier, Maya Henry, Regina Jokel, Lyndsey Nickels, Cathleen Taylor-Rubin. Semantic Variant Primary Progressive Aphasia: practical recommendations for treatment from 20 years of behavioural research. Brain Sciences, 2021.*

1.1. Thérapies visant le ré-apprentissage de mots et de concepts

Pour qui? Toutes les personnes atteintes de vsAPP capables de s'engager dans une thérapie (p.ex.,, sans troubles graves du comportement) sont candidates au traitement, quelle que soit la gravité de leur maladie. Les variantes de la vsAPP, avec atrophie dominante de l'hémisphère gauche ou de l'hémisphère droit, présentent des profils cliniques différents et la thérapie doit être adaptée en conséquence.

Quoi? Des gains thérapeutiques plus importants sont obtenus avec du matériel de traitement basé sur des mots/concepts qui sont personnellement familiers (p.ex.,, votre propre grille-pain au lieu d'un grille-pain standard), qui appartiennent au vocabulaire quotidien et qui ont des connaissances sémantiques résiduelles (c.-à.-d., que la personne peut encore comprendre le concept).

Comment?

- 20 à 60 min de pratique quotidienne (ou tous les deux jours), comprenant 15 à 30 items par session, devraient produire des gains visibles dès le premier mois de pratique ou avant;
- La thérapie visant l'anomie peut améliorer la récupération de mots dont la connaissance sémantique est préservée. Cependant, lorsque cette approche est utilisée avec des concepts pour lesquels les connaissances sémantiques sont dégradées, elle peut conduire à un apprentissage "par cœur" (rappel rigide de mots et dépendant du contexte). Pour améliorer les chances d'un apprentissage flexible et d'une généralisation de ces items, il est recommandé d'utiliser des stratégies qui ciblent la nature sémantique de l'anomie (p.ex., en exploitant la mémoire épisodique et autobiographique et les caractéristiques sémantiques, telles que l'utilisation et la localisation);
- La téléthérapie et les programmes auto-administrés à domicile se sont avérés faisables dans le cadre de la vsAPP. Les gains sont comparables à la thérapie en face à face.

Maintien

Les gains peuvent être maintenus jusqu'à 6 mois dans les cas légers (sans besoin de révision);

Une révision de faible intensité du vocabulaire ré-appris est conseillée tous les 2 mois pour les vsAPP légers-modérés, et toutes les 2–3 semaines pour les vsAPP sévères.

Généralisation

La généralisation reste limitée dans la vsAPP pour les items non entraînés. L'utilisation de mots entraînés en dehors du contexte de la thérapie a été rapportée de manière anecdotique dans certains cas. Le choix du programme thérapeutique peut influencer la généralisation.

Prophylaxie

a pratique régulière de mots/concepts que la personne peut encore nommer peut contribuer à ralentir la progression de la perte sémantique et de l'anomie pour ces éléments.

1.2. Technologies d'assistance et entraînement aux AVQ

Les technologies portables (p.ex., les dictionnaires visuels numériques), ainsi que l'entraînement aux AVQ avec l'utilisation de la mémoire procédurale, pourraient

favoriser l'indépendance dans la vie quotidienne des personnes atteintes de svPPA, comme le suggèrent les quelques études menées à ce jour.

1.3. Groupes de soutien et programmes éducatifs

Les groupes d'éducation et de soutien par les pairs sont des adjuvants précieux à la thérapie. Ils ont des effets bénéfiques sur la santé mentale, la gestion des symptômes de la maladie et la qualité de vie.

Supplementary 3 (Spanish). Variante semántica de la afasia progresiva primaria (APP-s): recomendaciones basadas en evidencia para el manejo terapéutico

Las siguientes recomendaciones clínicas se han elaborado de acuerdo con la mejor evidencia disponible en julio de 2021 como parte del documento: Aida Suárez-González, Sharon A. Savage, Nathalie Bier, Maya Henry, Regina Jokel, Lyndsey Nickels, Cathleen Taylor-Rubin. Semantic Variant Primary Progressive Aphasia: practical recommendations for treatment from 20 years of behavioural research. Brain Sciences, 2021.

1.1. Terapias de reaprendizaje de palabras y conceptos

¿A quién? Todas las personas con APP-s capaces de participar en la terapia (por ejemplo, sin alteraciones graves del comportamiento) son candidatas a tratamiento, independientemente de la gravedad de la enfermedad. Las variantes de APP-s con atrofia dominante del hemisferio izquierdo y derecho muestran perfiles clínicos diferentes y la terapia debe adaptarse en consecuencia.

¿Qué? Se obtienen mayores ganancias terapéuticas con material de tratamiento basado en palabras/conceptos familiares para la persona (por ejemplo, su propia tostadora en lugar de una tostadora estándar), que pertenezcan al vocabulario cotidiano y que retengan cierto significado semántico residual (es decir, que la persona todavía pueda entender el concepto).

¿Cómo?

- 20–60 minutos de práctica diaria (o en días alternos) con 15–30 palabras/conceptos por sesión deberían producir ganancias visibles en el primer mes de práctica o antes.
- La terapia para la anomia puede mejorar la recuperación de palabras con significado semántico conservado. Sin embargo, cuando este enfoque se utiliza con conceptos en los que el conocimiento semántico está degradado, puede conducir a un aprendizaje puramente memorístico (rígido y dependiente del contexto). Para mejorar las posibilidades de aprendizaje flexible y la generalización de estas palabras/conceptos, se recomiendan estrategias dirigidas a la naturaleza semántica de la anomia (por ejemplo, aprovechando la memoria episódica y autobiográfica y las características semánticas del concepto como el uso y la ubicación).
- La teleterapia y las terapias autoadministradas en casa han demostrado ser factibles en la APP-s. Los beneficios terapéuticos son comparables a los de la terapia presencial.

Mantenimiento

Las ganancias terapéuticas pueden mantenerse hasta 6 meses en los casos leves (sin necesidad de revisión)

Se aconseja una revisión de baja intensidad del vocabulario reaprendido cada 2 meses para la APP-s leve-moderada y cada 2–3 semanas para la APP-s grave.

Generalización

La generalización sigue siendo limitada en la APP-s para los elementos no entrenados. Anecdóticamente se han descrito casos del uso de palabras entrenadas fuera del contexto de la terapia. La elección del tipo de técnica terapéutica puede influir en los resultados de generalización.

Profilaxis

La práctica regular de palabras/conceptos que la persona todavía puede nombrar podría ayudar a frenar la progresión de la pérdida semántica y la anomia para dichas palabras/conceptos.

1.2. Tecnologías de asistencia y entrenamiento de las AVD

Las tecnologías portátiles y el entrenamiento de las AVD podrían favorecer la independencia en la vida diaria de las personas con APP-s, tal y como sugieren los pocos estudios realizados hasta la fecha.

1.3. Grupos de apoyo y programas educativos

Los grupos educativos y de apoyo de pares son valiosos coadyuvantes de la terapia, aportando beneficios para la salud mental, el manejo de los síntomas de la enfermedad y la calidad de vida de las personas con APP-s.
